# Echinacoside Attenuates LPS-induced Neuroinflammation and Ameliorates Memory Deficit by Modulating the Microbiota-Gut-Brain Axis

**DOI:** 10.4014/jmb.2605.05043

**Published:** 2026-07-30

**Authors:** YiXi Zeng, Yanqing Ma, Shunfan Chen, Lanyue Zhang, Hui Li

**Affiliations:** 1 School of Biomedical & Pharmaceutical Sciences, Guangdong Provincial Key Laboratory of Plant Resources Biorefinery, Guangdong University of Technology, Guangzhou 510006, P. R. China; 2 Guangdong Provincial People’s Hospital, Guangdong Academy of Medical Sciences, Guangdong Geriatric Institute, Guangzhou 510220, P. R. China

**Keywords:** *Cistanche tubulosa* (Schenk) wight, Echinacoside, Inflammation, Intestinal flora, Microbiota-Gut-Brain axis

## Abstract

We aimed to investigate the neuroprotective effect of Echinacoside (ECH) extracted from *Cistanche tubulosa* (Schenk) Wight in a mouse model of LPS-induced neuroinflammation and associated memory impairment, and the potential role of the microbiota-gut-brain axis (MGBA) in this process. Intraperitoneal injection of LPS in mice can induce inflammation, causing learning and memory impairment, and it can also affect the balance of the intestinal flora. The Morris water maze (MWM) experiment, immunohistochemistry, immunofluorescence chemistry, Nissl staining, and 16S rRNA gene sequencing of the mice intestinal flora were performed for the study. The results of the MWM experiment showed that mice treated with ECH were able to find the platform faster compared to the model group, with shorter distances moved than the *C. tubulosa* (Schenk) Wight extract (CTE) group, and they had more platform crossings. Immunofluorescence staining of brain cortex and hippocampal tissues showed that ECH could attenuate LPS-induced neuronal damage and reduce the expression levels of Ionized calcium-binding adapter molecule 1 (Iba-1) positive cells and the Tumor Necrosis Factor-alpha (TNF-α). 16S rRNA gene sequencing of mice intestinal flora revealed that ECH exerted beneficial effects by reshaping the structure of the intestinal flora. Our findings suggest that ECH may be beneficial for alleviating neuroinflammation-related cognitive impairments, providing theoretical support for a new neuro-psycho-pharmacological approach to MGBA-related diseases through restoring intestinal flora homeostasis.

## Introduction

Cognitive impairment refers to cognitive functional abnormalities, with symptoms including memory issues, spatial disorientation, executive dysfunction, communication difficulties, and emotional changes [1, 2]. With advances in research on memory deficit diseases, neuroinflammation is currently considered to be one of the primary drivers. Although significant breakthroughs have been made in the research on the pathophysiological mechanisms of neuroinflammation, there is still a lack of effective preventive and treatment measures to alleviate cognitive impairment in clinical practice. Recent studies have emphasized the potential role of the gut microbiome in regulating central nervous system function via the microbiota-gut-brain axis (MGBA) [3]. The MGBA, an interactive network connecting the gut and the brain, comprises neural, endocrine, and immune pathways. Neurally, the MGBA includes neural pathways such as the vagus nerve, which enable bidirectional information transmission between the gut and the brain. This neural pathway can influencing emotions, cognitive functions, and behaviors. Recent research has identified a substantial link between neurological diseases and gut fungi [4]. However, due to its complexity, research on complex diseases, particularly on how the MGBA is related to and influences the pathogenesis of neurocognitive disorders, remains incomplete.

*Cistanche tubulosa* (Schenk) Wight is a common traditional Chinese medicine, known as the “desert ginseng” and has excellent tonic effects. Plants of the genus Cistanche (family Orobanchaceae) are perennial parasitic medicinal plants. It is widely used in traditional Chinese medicine and folk therapies and has various medicinal properties. It can be used to enhance the immune system, improve kidney function, enhance sexual function, delay aging, treat impotence, chronic kidney disease, female infertility, excessive bleeding, pathological leukorrhea, senile constipation and other diseases [5-8]. Among the various active ingredients of *C. tubulosa*, the content of phenylethanoid glycosides is the highest, and Echinacoside (ECH) is one of the components with a relatively high content among the phenylethanoid glycosides of *C. tubulosa*, has strong biological activity and good development and application prospects. Previous research has demonstrated that ECH exhibits a variety of pharmacological activities, including antioxidative effects [9], neuroprotection [10], anti-inflammatory properties, liver protection, and anti-tumor effects [11], among others.

This study aims to explore the neuroprotective effect of ECH on lipopolysaccharide (LPS)-induced learning and memory impairment in C57BL/6 mice. The potential involvement of the microbiota-gut-brain axis is investigated through the Morris Water Maze (MWM) experiment, immunohistochemistry, immunofluorescence chemistry, Nissl staining, and 16S rRNA gene sequencing of the mouse gut microbiota. The protective effect of ECH on LPS-challenged neurons and its beneficial regulation of the gut microbiota structure are verified, providing theoretical support for new treatment strategies for neuroinflammation-related cognitive disorders.

## Materials and Methods

Tong Ren Tang Company provided dried rhizome of *C. tubulosa* (China). Professor Nian Liu identified all the plant samples (Zhongkai University of Agriculture and Engineering, Guangzhou, China). Some of them were kept as voucher specimens at Guangdong University of Technology's Institute of Natural Medicine and Green Chemistry in Guangzhou, China (No. 2018-116-C). Aladdin Reagent Inc. provided all the chemicals used in this study (China). All chemicals were of analytical grade.

### Isolation of Echinacoside

**Fig. 1:**
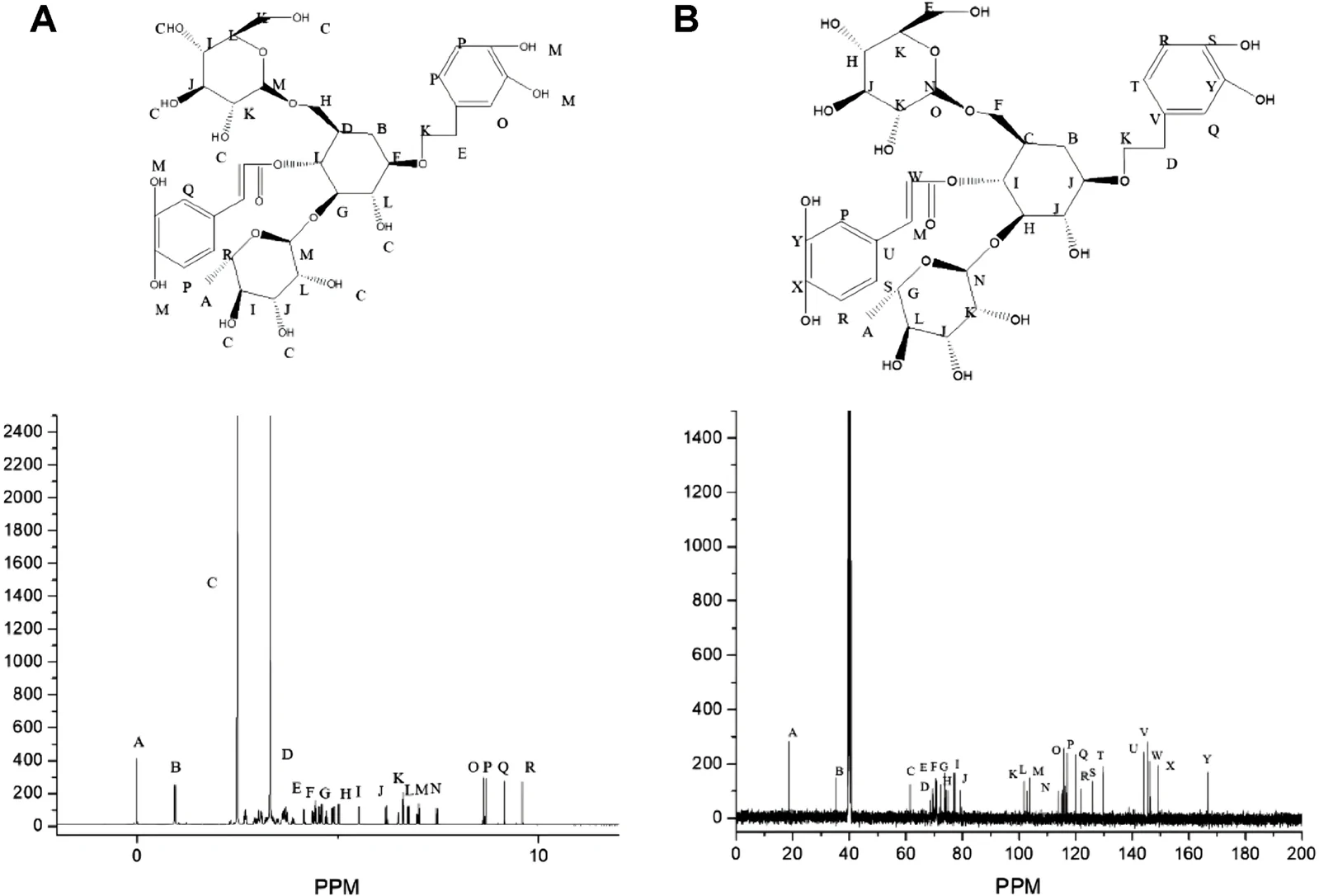
NMR analysis of Echinacoside. (**A**) ^1^H NMR. (**B**) ^13^C NMR

The *C. tubulosa* powder (100 mg) was dissolved in 5 mL of distilled water and purified using a Sephadex LH-20 column (100 mL; GE Healthcare Bio-Sciences AB, Sweden), which was eluted with a 10% methanol solution, and monitored using HPLC. Fractions containing phenylethanoid glycosides were monitored by absorbance at 245 nm and collected using an automatic sampler. After silica gel (200-300 mesh) column chromatography, ECH was eluted with petroleum ether (PE) at first, then PE/ethyl acetate (EA) (100:1) and EA finally. Preparative HPLC was performed using Agilent1260 instrument which was equipped with DAD detector and COSMOSIL RP-18 column (5 μm, 20 mm I.D. × 250 mm; flow rate: 6 mL/min; 25°C; detection wavelength: 280 nm; TR 31.5 min). MeOH-H2O (80: 20) was used as an eluent. About 970 mg of ECH was obtained (HR-ESI-MS and NMR, Fig. 1) [12]. The purity of the isolated ECH was determined to be 97.2% by HPLC analysis (area normalization method), confirming its suitability for in vivo pharmacological studies.

### Animals and Treatment

A total of forty-two SPF-grade male C57BL/6 mice (aged 7−8 weeks, with a weight range of 18−22 g) were purchased from the Animal Center of Sun Yat-Sen University (SCXK2011-0029, China). All mice were acclimatized in a pathogen-free animal room maintained at a controlled temperature of 23 ± 2°C, with a 12/12-hour light/dark cycle. They were provided with standard laboratory diet and water. The mice were allowed to acclimatize to the facility for one month prior to the initiation of experimental procedures. All animals were looked after and conducted experiment followed the guidelines set by the National Institutes of Health (the 7th ed., USA). All animal procedures were performed in accordance with the National Institutes of Health Guide for the Care and Use of Laboratory Animals (7th ed.) and were approved by the Institutional Animal Care and Use Committee of Guangdong University of Technology (Approval No. GDUTXS20250184). Prior to blood collection and tissue harvesting, mice were deeply anesthetized via intraperitoneal injection of sodium pentobarbital (50 mg/kg body weight). The depth of anesthesia was confirmed by the absence of pedal withdrawal reflex before proceeding with cervical dislocation for euthanasia. Mice were randomly divided into following seven groups (Six mice per group): Control group, LPS group, TTP488 positive group, CTE group, ECH-L group, ECH-M group, ECH-H group. Except for the control group, mice in all other groups were injected with 250 μg/kg of LPS solution (diluted in sterile saline, 100 μL/10 g) before drug treatment to induce a mouse neuroinflammation model. Injections were given once daily for seven consecutive days. The control group was injected with the same volume of sterile saline for seven days. After modeling, the control group and LPS group were orally gavaged with sterile saline (100 μL/10 g) for 7 days; the TTP488 positive group was orally gavaged with 5 mg/kg TTP488 solution daily for 7 days (diluted in sterile saline, 100 μL/10 g); the CTE group: orally gavaged with 50 mg/kg *C. tubulosa* extract solution daily for 7 days (diluted in sterile saline, 100 μL/10 g); ECH-L group: orally gavaged with 25 mg/kg ECH aqueous solution daily for 7 days (diluted in sterile saline, 100 μL/ 10 g); ECH-M group: orally gavaged with 50 mg/kg ECH aqueous solution daily for 7 days (diluted in sterile saline, 100 μL/10 g); ECH-H group: orally gavaged with 100 mg/kg ECH aqueous solution daily for 7 days (diluted in sterile saline, 100 μL/10 g). The doses of ECH (25, 50, and 100 mg/kg) were selected based on our preliminary dose-finding experiments (data not shown) and previous reports demonstrating neuroprotective effects of ECH at similar dosage ranges in rodent models of cognitive impairment.

### Morris Water Maze Test (MWM)

On the 8th day after drug treatment, the Morris water maze experiment was initiated to start modeling. The water maze consisted of a dark stage (10 cm in diameter), and nontransparent water at 24 ± 1°C in a pool (120 cm in diameter). The movements of the mice were tracked, and all experiments were recorded using a camera overhead which was connected to an Intelligent Video Tracking Analysis System (TSE, Germany). The platform (1 cm underwater) remained in the middle of the target quadrant during navigation tests (the target quadrant). There were four training trials (once a day). Each experiment lasted for 60 s unless the mice climbed up to the platform, and the time period of escape was recorded. During the testing phase (day 12), starting at 14:00, the escape latency of mice entering the water from different quadrants was recorded. The indoor environment was kept quiet, and personnel movement was minimized. During this phase, regardless of whether the mice found the survival platform within 1 minute, they were not required to stay on the platform. The spatial exploration experiment (day 13) began at 14:00. The survival platform was removed, and the mice were placed in the quadrant diagonally opposite to the original platform location. The computer system recorded the number of times the mice entered the original platform quadrant within 1 minute and their movement trajectories. The space probe test was performed in the platelet quadrant following the navigation test. Each mouse swam for 60 s after being placed into a pool. The system was used to measure the percent time that the mice took to cross the platform area, and the time they spent in each quadrant [13-15]. On the 14th day, the mice were euthanized by cervical dislocation, and brain tissue samples were collected.

### Dissection and Tissue Collection

After blood collection, the mice are euthanized by cervical dislocation. Alcohol at 75% concentration is sprayed on the head of the mouse to disinfect and moisten the fur, preventing hair from sticking to the tissue. The head skin is quickly cut open, and with surgical scissors, a careful incision is made from the midline and sides of the skull. Tweezers are used to gently remove the cut skull, and then the entire brain tissue is extracted. It is placed in a large dish containing pre-chilled saline to wash away excess blood and hair. Excess water is removed by blotting with absorbent paper. The left half of the brain tissue is taken for fixation in an Eppendorf tube containing 4% paraformaldehyde, while the right half is placed in a cryotube, labeled, and promptly stored in liquid nitrogen before transferring to a -80°C freezer for long-term preservation. Collect fecal samples and store them at -80°C for further analysis [16].

### Nissl Staining

Each group of control tissue samples and the experimental tissue samples were washed two times for 5 10 min with 0.01 M PBS. The researchers used a rotating microtome to cut 5 μm thick coronal sections from paraffin-embedded brain specimens until they reached the hippocampal surface. For hippocampal analysis, images were captured from the CA1 region at 400× magnification, with three randomly selected non-overlapping fields per section. The CA1 region was selected due to its well-established role in spatial memory processing. The sections were stained with 0.5% cresyl violet solution for 10 min. After that, they were rinsed with distilled water, and dehydrated with a range of graded ethanol solutions (70, 95, and 100%) at last. After washing in xylene for 5 min, the slides were mounted with mounting medium. Normal neurons were observed and photographed under a microscope at 400 times magnification. Each slice contained three randomly chosen non-overlapping areas. Nissl particles are distinct in normal neurons, and the nucleus is light and clear. The amount of normal neurons was counted. And then the mean value was calculated to analysis [17].

### Immunofluorescence Assays

After drying overnight, slices were washed three times in 0.01 M PBS for 5 min each time. For hippocampal analysis, images were captured from the CA1 region at 400× magnification, with three randomly selected non-overlapping fields per section. The CA1 region was selected due to its well-established role in spatial memory processing. The sections were blocked with BSA, and put them on a shaker at room temperature for 1 h. Primary antibody (Iba-1, 1:200, GB13105-1) was incubated overnight at 4°C on samples, followed by a 1 h incubation at room temperature with secondary antibody (Iba-1, 1:200, GB13105-1) (anti-rabbit, 1:300, GB21303). The slices were stained with DAPI (300 nM, G1012) for nuclear counterstaining. A Nikon confocal microscope was used to capture the images [18].

### Immunohistochemistry Staining

The following procedure was used to express TNF-α in the brain in situ: Serial 5 μm tissue slices were put in xylene (twice for 5 min at ambient temperature), 100% ethanol (twice for 2 min at room temperature), 95% ethanol (for 1 min), and 80% ethanol (for 1 min). For hippocampal analysis, images were captured from the CA1 region at 400× magnification, with three randomly selected non-overlapping fields per section. The CA1 region was selected due to its well-established role in spatial memory processing. Antigen retrieval was accomplished by boiling the slides in 1 mM EDTA solution (pH 8.0) (for 30 min). Slides were blocked with 1% FBS and 0.4% TritonX-100 at room temperature (for 1 h), followed by incubation with primary anti-TNF antibody overnight at 4°C. The sections were incubated with goat anti-rabbit antibody after washing three times with blocking buffer. To stain the nuclei, sections were counterstained with hematoxylin for (2 min). A microscope was used to examine the sections that were labeled to detect TNF-α-positive cells [9].

### Operational Taxonomic Units (OTU) and Statistical Analyses

From PCR-amplified 16S rRNA genes, Trimmomatic was used to inspect and filter the generated sequences. The reads were clustered into OTUs using DNACLUST, Cumulative Sum Scaling was used to normalize them, and metagenomeSeq was used to assess the differential abundance. Using QIIME2, longitudinal (Qiime longitudinal pairwise differences), volatility (Qiime longitudinal volatility), and alpha and beta diversity analysis (Qiime diversity core-metrics) were carried out. Analysis of log-fold changes and inspection of features were conducted using the Metaviz platform, and Krona and QIIME2 were used to create interactive relative abundance plots. Both nonparametric Wilcoxon rank sum testing, and metagenomic sequencing were used to find significant difference in relative abundance at the species level between paired samples statistically [19].

### Statistical Analysis

In this study, one-way analysis of variance (ANOVA) was used for statistical analysis. Following one-way ANOVA, post hoc multiple comparisons among the seven groups were performed using Tukey's honestly significant difference (HSD) test, which controls for family-wise error rate. Normality was assessed using the Shapiro–Wilk test. The homogeneity of variance of the data was verified by the Brown-Forsythe test. The results confirmed that the data met this condition and the requirements of normality, ensuring the accuracy of statistical inference and interpretation. Among them, *p* < 0.05, *p* < 0.01, and *p* < 0.001 indicate significant, highly significant, and extremely significant differences compared with the LPS group, respectively.

For the 16S rRNA sequencing data, genus-level differential abundance analysis was performed using the Kruskal–Wallis H test as an exploratory screening approach to identify candidate taxa potentially associated with ECH treatment. We acknowledge that this approach, without correction for multiple testing, may increase the risk of type I errors. Therefore, all p-values from the microbiome analysis are reported uncorrected and should be interpreted as hypothesis-generating rather than confirmatory, warranting further validation in future studies.

## Results

### Echinacoside improved LPS-Induced Memory Loss

**Fig. 2:**
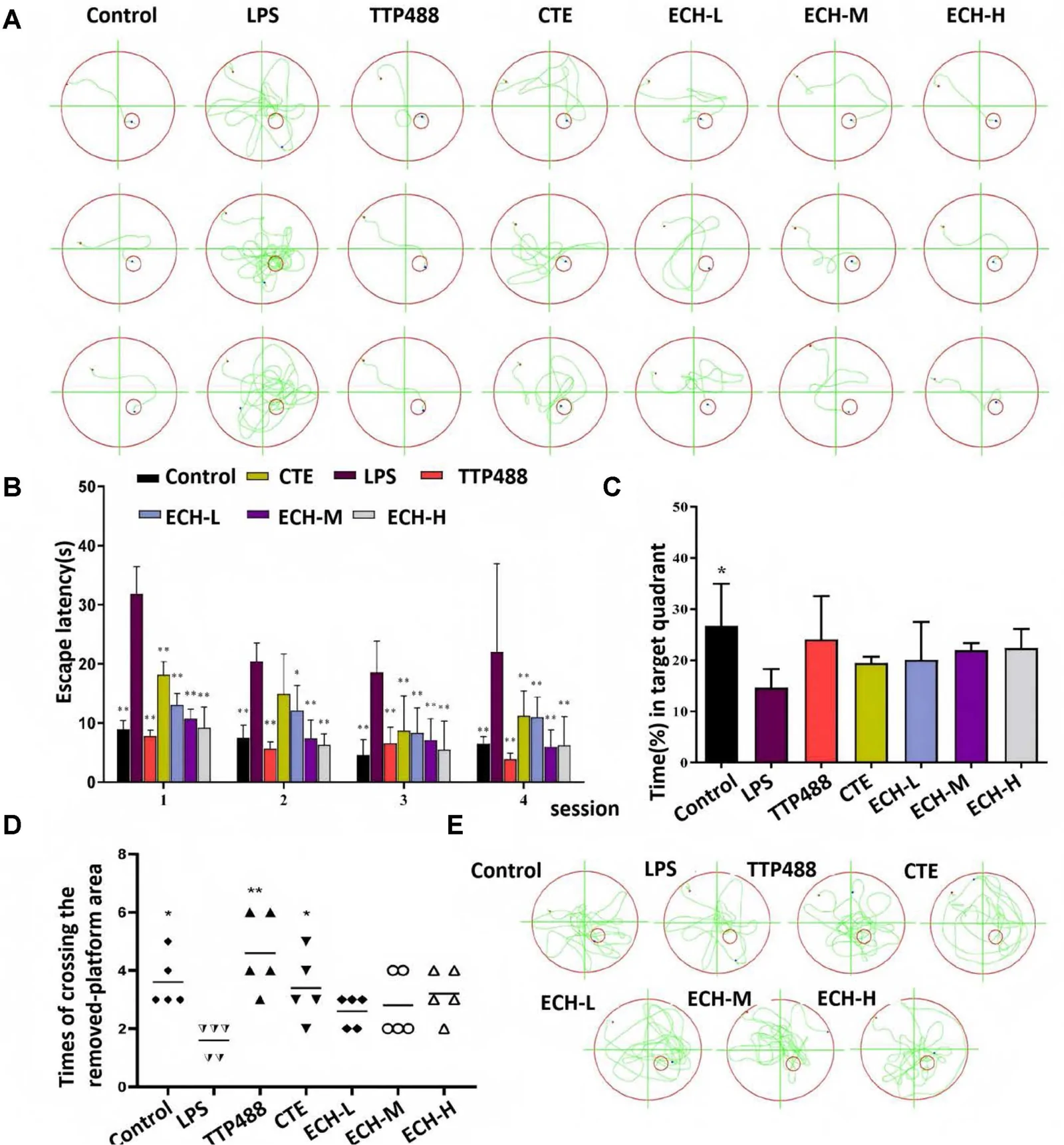
Effects of Echinacoside on LPS-induced memory impairment. (**A**) Movement of mice in the place-navigation (hidden-platform) test. (**B**) Position navigation (hidden platform) to test mouse escape latency. (**C**) The percentage of time mice spent seeking the target quadrant in the spatial probe test. (**D**) The number of times mice traversed the removed-platform territory in the spatial probe test. (**E**) Movement trails of mice in the spatial probe test. The differences were considered significant at *p < 0.05 and **p < 0.01.

LPS injection in mice is known to cause deficits in memory and learning [20, 21]. Using MWM, the effects of ECH on memory and learning in LPS-injected mice were investigated. The navigation test was conducted with the platform obscured (Fig. 2B), and experimental data were examined. The avoidance latency of mice in the LPS group (t(8) = 4.264, *p* = 0.0027) was longer in the first, second, third, and fourth stages than that of mice in the control group. The escape latency of LPS mice during the first, second (*p* < 0.05), and third (*p* < 0.01) sessions was reduced by ECH. The CTE group's escape latencies were considerably longer than the ECH-L, ECH-M, and ECH-H groups. The path taken by the mice to the platform was consistent with the escape incubation period, but the ECH-treated groups travelled shorter distances (Fig. 2A).

The platform was removed using a spatial probe test. It was significant to discover that the control group spent less time in the target quadrant compare with the LPS group, and the mice also crossed over to the platform area in significantly less time. ECH-treated mice spent more time in the target quadrant (Fig. 2C). In addition, ECH-treated mice crossed the plateau area more times than the LPS group (Fig. 2D). The tracks of the mice were recorded, and the trajectory followed by the mice was found to be consistent with the escape incubation period (Fig. 2E).

### Echinacoside Attenuated LPS-Elicited Neuronal Injury

**Fig. 3:**
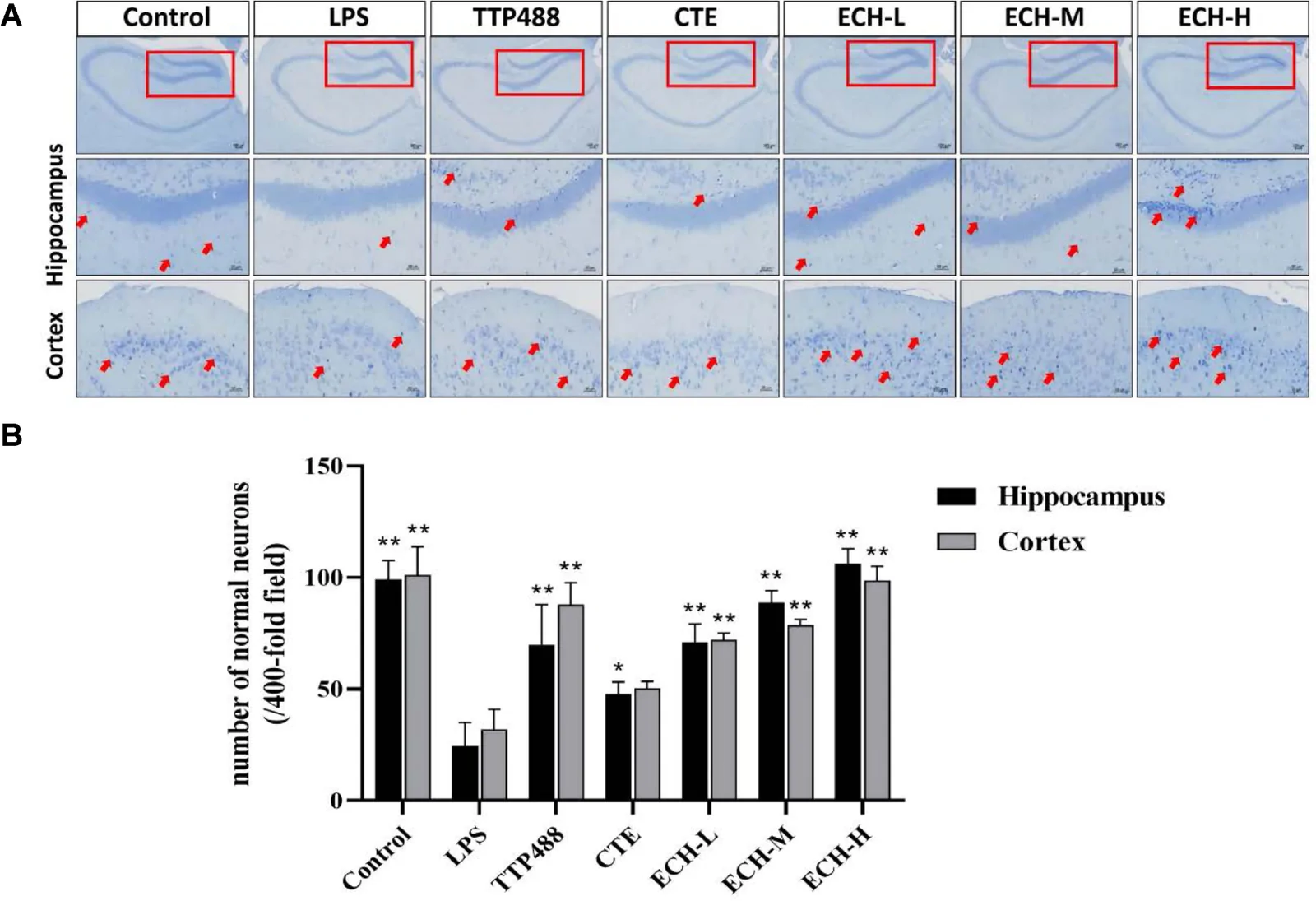
Influence of Echinacoside on LPS-elicited neuronal injury. (**A**) Nissl staining and normal neuronal counts in hippocampus and cortex of each group; (**B**) Number of normal neurons. Data represent the mean ± SD (n = 6). Significance of differences: **p < 0.01.

Histopathological changes were observed by Nissl staining to evaluate the protective effect of ECH on neurons. As indicated by the red arrow, the Nissl staining shows a significant loss of Nissl substance in the hippocampus (F(6, 36) = 7.025; *p* = 0.0001) and cortex (F(6, 36) = 3.005; *p* = 0.0214) regions in the LPS-treated group, whereas the content of Nissl substance in the control group was found to be higher. ECH treatment significantly restored Nissl substance content and increased the number of morphologically normal neurons compared to the LPS group, indicating attenuation of LPS-induced neuronal injury (Fig. 3) [22]. TTP488 treatment exerts neuroprotective effects by inhibiting the RAGE receptor, reducing Aβ plaque deposition, and decreasing the production of inflammatory cytokines [23]. ECH demonstrates comparable neuroprotective effects to TTP488, with superior outcomes compared to the CTE group, and exhibits a concentration-dependent effect.

### Echinacoside Iinhibited LPS-Induced Inflammation in the Cerebral Cortex and Hippocampus of C57BL/6 Mice

**Fig. 4:**
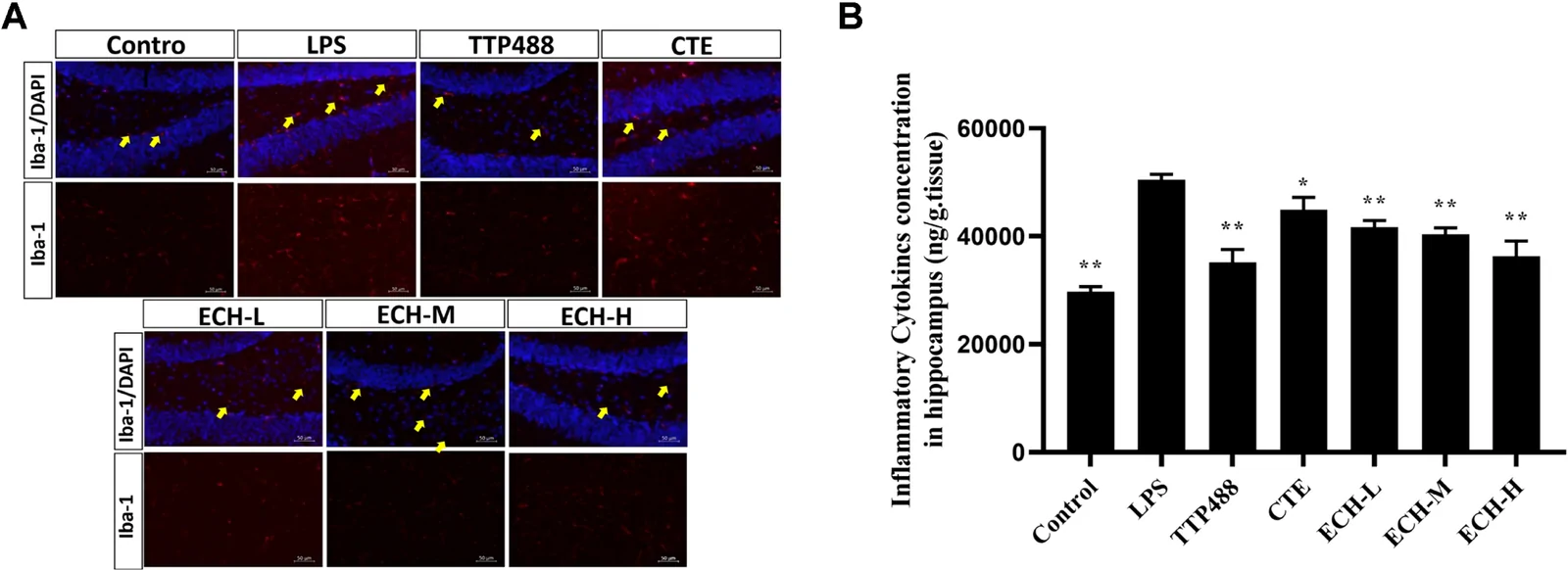
The effect of Echinacoside on LPS-induced inflammatory response in the hippocampus of C57BL/6 mice. (**A**) Immunofluorescence analysis of microglia (Iba-1-positive cells) in the experimental groups, scale bar = 50 μm. (**B**) inflammatory cytokine concentration in hippocampus of mice brain. Data indicate the mean SD (n = 6 mice per group). The prominence of differences from the LPS group is at **p < 0.01.

**Fig. 5:**
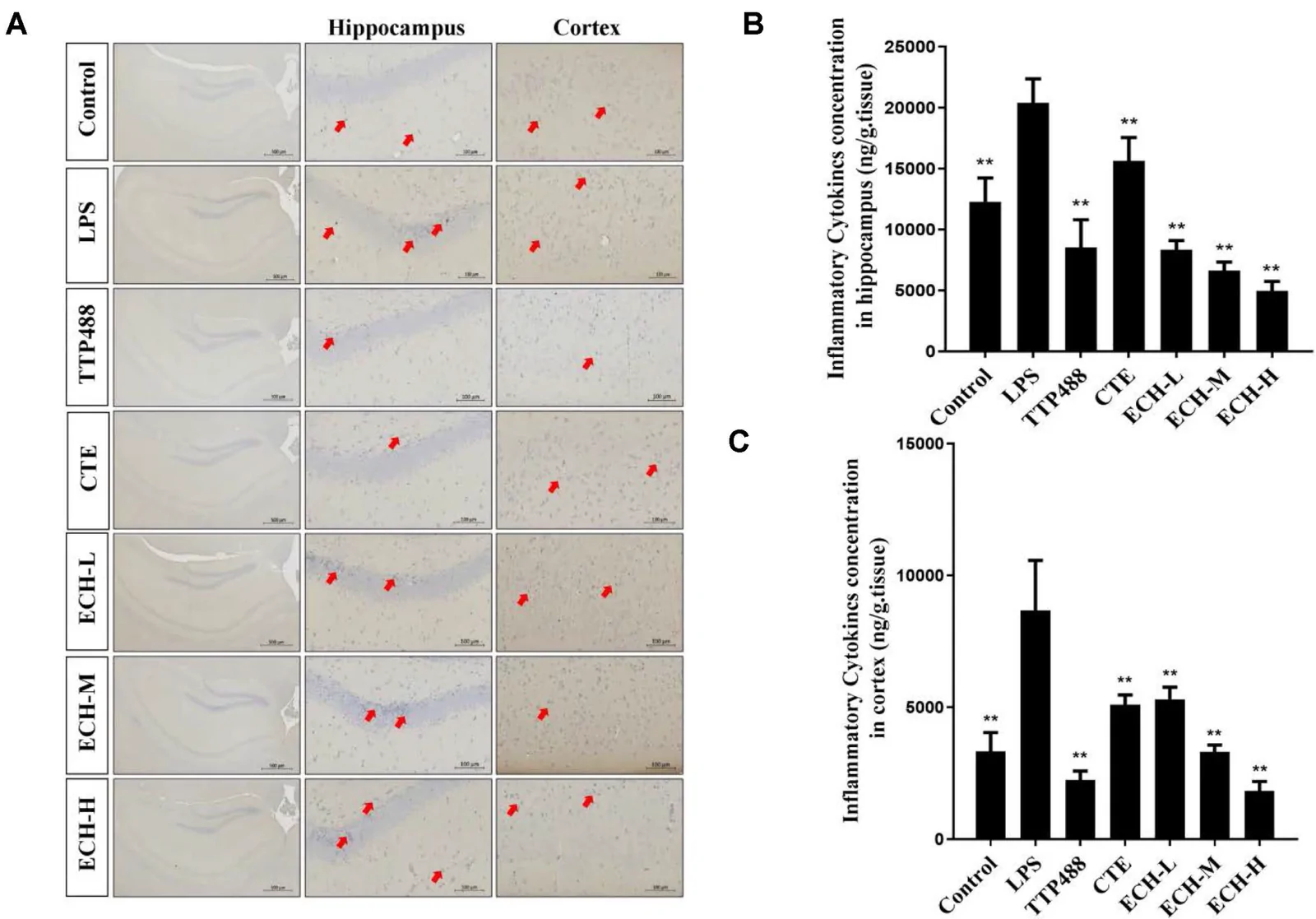
Inhibitory effect of Echinacoside on LPS-induced inflammatory response in cortex and hippocampus of C57BL/6 mice. (**A**) Immunostaining of TNF-α in the cortex and hippocampus. (**B**) Integrated Optical Density in the hippocampus of mice brain. (**C**) Integrated Optical Density in the cortex of mice brain. Data indicate the mean SD (n=6 mice per group). Significant differences: *p < 0.05 and **p < 0.01.

Neuronal damage and cognitive deficits result from the hyperactivation of microglia and the action of cytokines [24]. Therefore, we investigated the effects of ECH on inhibition of LPS-induced microglial activation. Confocal microscopy showed that intraperitoneal injection of LPS increased the number of activated glial cells (Iba-1 reactive cells) in adult mice, while ECH treatment decreased the number of such cells in the LPS-induced group (Fig. 4) (F (6, 36) = 41.29; *p* < 0.0001). Moreover, immunohistochemical imaging showed an increase in TNF-α expression level in the cortex and hippocampus of mice who received intraperitoneal injection of LPS. However, ECH-M and ECH-H effectively reduced TNF-α expression (Fig. 5) (F (6, 36) = 2.406; *p* = 0.0473). These findings suggest that ECH treatment can help in reducing the inflammatory response in the brain of the mouse, thus preventing further neuronal damage, and improving cognitive ability.

### Echinacoside Alter Gut Microbiota of C57BL/6 Mice

**Fig. 6:**
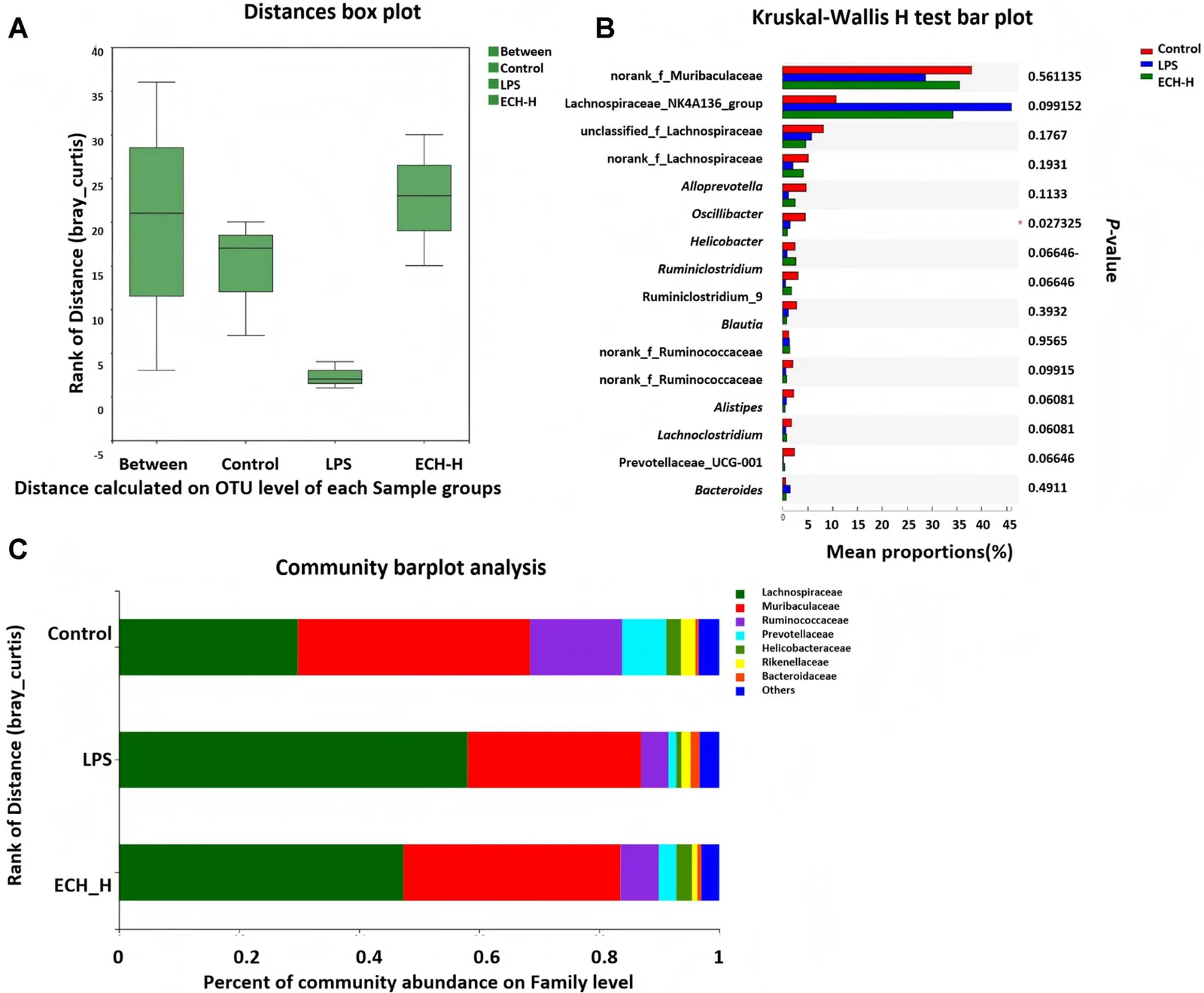
Response of gut microbiome to Echinacoside treatment in LPS-induced inflammatory mice. (**A**) ANOSIM revealed significant differences in gut microbiota structure (Bray-Curtis ANOSIM = 0.3909) among different groups. (**B**) Relative abundances of genera that showed significant differences among samples from gut microbiota. The significancse of differences between the groups was determined using a one-way ANOVA (*P < 0.05; **P < 0.01). (**C**) The composition of the gut microbiome in different groups at the family level. Within each community, the relative read abundances of different bacterial genera are shown. “Others” includes taxa with an abundance of less than 1%.

Based on the Venn diagram analysis, the OTU number in the LPS group was significantly higher than that in the drug groups, indicating that LPS injection altered the intestinal flora diversity. The intestinal contents of mice in the LPS group, showed unique flora suggesting that LPS treatment can also change the colony type of the sample. The level data of bacterial genera were analyzed using principal component analysis (PCA), and the similarity of the intestinal flora in various groups was assessed using a multivariate statistical approach. According to PCA's findings (Fig. 6), the bacterial structure of the LPS-producing bacterial colonies in the control group was significantly different from that of the ECH-H treatment group.

### Echinacoside Modulates Gut Microbiota Diversity and Composition

To further characterize the impact of ECH on the gut microbial community, we evaluated both alpha and beta diversity indices. Alpha diversity analysis using the Shannon index (reflecting community evenness) and Chao1 index (reflecting species richness) revealed that LPS treatment significantly reduced gut microbial diversity compared to the control group (*p* < 0.05 for both indices). ECH-H treatment significantly restored Shannon diversity (*p* < 0.05) and Chao1 richness (*p* < 0.01) compared to the LPS group, indicating a partial recovery of the gut microbial community complexity. Beta diversity analysis using principal coordinate analysis (PCoA) based on Bray-Curtis distances demonstrated distinct clustering patterns among groups. Specifically, the ECH-H group clustered towards the control group and was clearly separated from the LPS group (PERMANOVA, *p* < 0.01). These results indicate that ECH treatment partially restores the gut microbial diversity and community structure disrupted by LPS, further supporting the modulatory role of ECH on the gut microbiota.

### Analysis of Changes in Intestinal Flora after Echinacoside Treatment

**Fig. 7:**
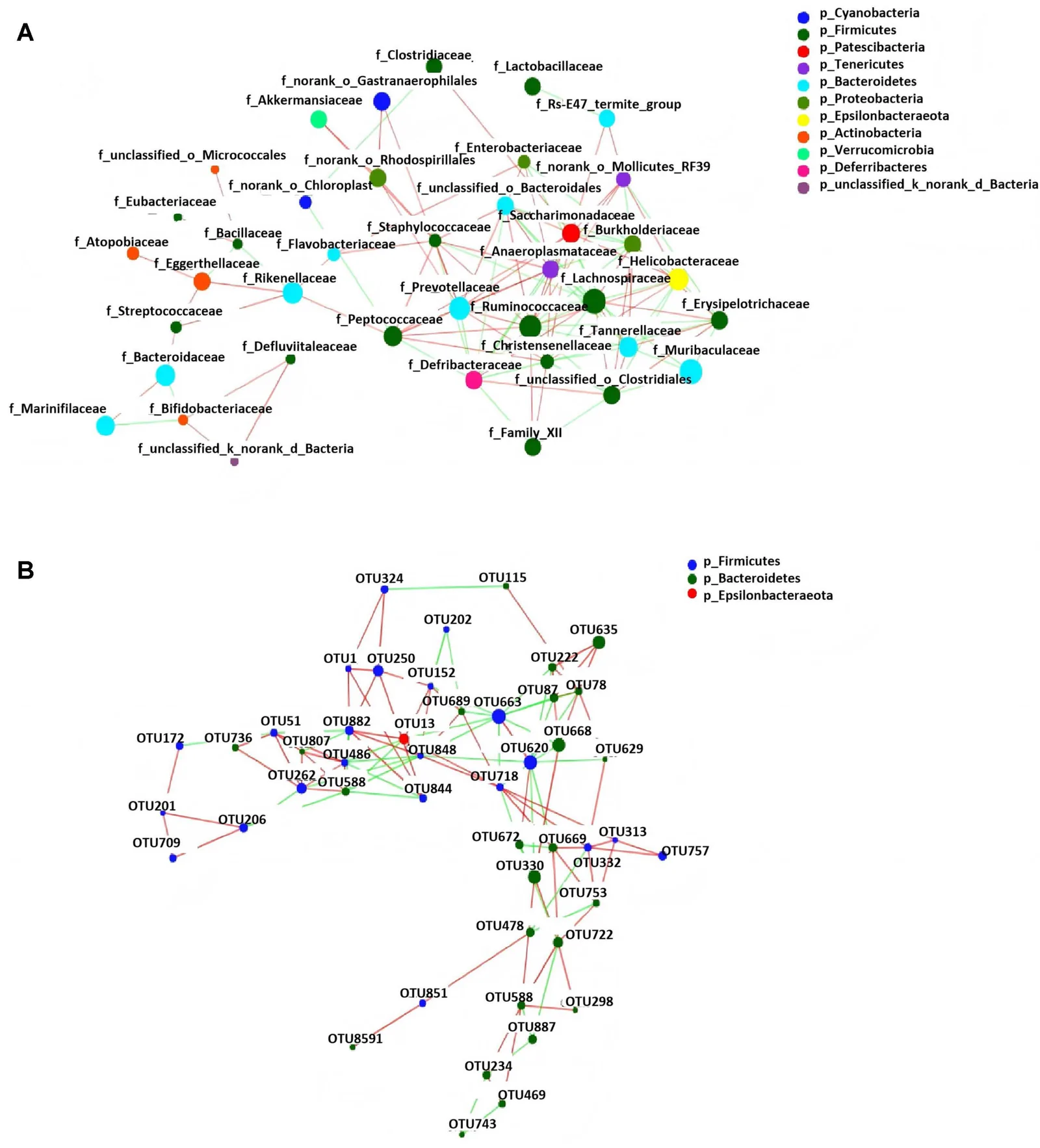
Co-occurrence analysis of the gut microbiome response to Echinacoside treatment in mice with LPS-induced inflammation. Co-occurring bacteria at the phylum level in the caecum (**A**) and colon (**B**). The relative abundance of the gut microbiota and heritability estimates are represented by the size and color of the nodes, respectively. Positive and negative correlations are indicated by red and green lines, respectively. The correlation's strength is reflected in the width.

ANOSIM analysis was used to determine whether intergroup differences were significantly greater than intragroup differences. Adonis analysis is used to examine the extent to which different grouping factors explain the sample differences. According to the intestinal content abundance data of mice in each group, species with different abundance differences in different microbial communities were detected using strict statistical methods, and hypothesis testing was conducted to intuitively identify different species. ANOSIM revealed significant differences in the interblock (Fig. 6; Bray-Curtis ANOSIM = 0.3909) of the gut microbiota among different mouse groups. The intestinal flora of the ECH group was significantly different from that of the LPS group, and the community composition was close to that of the control group. A correlation network diagram was used to study the relationship between different groups of intestinal flora. The horizontal correlation network map of the genera revealed a significant interaction between the different genera of intestinal flora (Fig. 7). There were 391 OTUs in the tissue samples of the control and LPS groups (26 +365 = 391), while 501 OTUs (365 + 136 = 501) were there in the tissue samples of the control and ECH groups. The results showed that the microbial composition of the control and ECH tissue samples was quite similar, suggesting that the treatment effect of ECH was better. PCA was used to analyze the difference in the composition of intestinal content among samples from control, ECH-H, and LPS mice. The results showed that the intestinal microbial composition of mice treated with ECH-H was significantly different from that of mice treated with LPS. The dilution of the PC1 axis was 22.12%, while that of the PC2 axis was 18.01%.

To further examine taxa potentially linked to neuroprotection, we extracted and compared the relative abundances of Lachnospiraceae and Muribaculaceae across groups. As shown in Fig. 6B, Kruskal-Wallis H test revealed that Lachnospiraceae_NK4A136_group (*p* = 0.09915), unclassified_f_Lachnospiraceae (*p* = 0.1767), and *Lachnoclostridium* (*p* = 0.06081) all exhibited higher relative abundances in the ECH-H group compared to the LPS group, indicating a restoration trend. Notably, *Oscillibacter* (*p* = 0.02732) showed the most prominent difference among groups, suggesting a potential association with ECH treatment that warrants further investigation. Meanwhile, Muribaculaceae (nonrank_f_Muribaculaceae) displayed a mean proportion in the ECH-H group approaching the control level (Fig. 6C). Community barplot analysis (Fig. 6C) visually confirmed that ECH-H treatment partially restored the proportions of both families, with their relative abundances converging toward control levels. These results suggest that ECH specifically modulates these two families, which are closely linked to short-chain fatty acid production and neuroimmune regulation.

## Discussion

This study primarily investigated the therapeutic efficacy of ECH, a major active component in the extract of *C. tubulosa*, in neuroinflammation, and linked it to the improvement of the gut microbiota environment through the gut-brain axis. Our experimental results demonstrated that ECH exerts significant neuroprotective effects in the LPS-induced neuroinflammation model, as evidenced by improved cognitive performance, reduced neuronal injury, and attenuated microglial activation.

Firstly, we focused on the impact of ECH on cognitive function and neuronal damage in mice. The research findings revealed that long-term high-dose ECH treatment significantly and dose-dependently restored learning and memory abilities in mice injected with LPS, with efficacy comparable to that of the positive control drug TTP488. Further analysis showed that ECH mitigated neuronal injury in the hippocampus and cerebral cortex, as evidenced by increased Nissl body content and normal neuronal counts, and attenuated neuroinflammatory responses by reducing the number of microglial positive cells (Iba-1) and the secretion of the inflammatory cytokine TNF-α. This finding aligns with the research results reported by [25] and [26], who respectively documented the alleviation of cognitive decline in SAMP8 mice and the improvement of neurobehavioral performance in mice with Parkinson's disease (PD) by ECH. Collectively, these studies indicate that ECH possesses remarkable neuroprotective effects, potentially mediated through the inhibition of neuroinflammatory responses.

Secondly, we explored the impact of ECH on the gut microbiota. Through analysis of the gut microbiota composition in mice, we found that following ECH treatment, the gut microbiota, diversity, and bacterial counts in mice were more similar to those in the Control group and the TTP488 group. This result aligns with previous studies indicating a regulatory relationship between the brain and the gut microbiota. Specifically, 16S rRNA sequencing revealed that ECH-H treatment significantly altered the abundance of *Oscillibacter* (*p* = 0.02732), with several other genera including *Alistipes* (*p* = 0.06031) and *Lachnoclostridium* (*p* = 0.06081) showing trends toward significant differences. Although Lachnospiraceae and Muribaculaceae did not reach statistical significance in our dataset, their relative abundances in the ECH-H group showed trends converging toward control levels (Fig. 6C). These two bacterial families play crucial roles in gut health and nervous system function. Lachnospiraceae is a well-known butyrate-producing family, and species such as *Ruminococcus_torques*_group and *Bacteroides* are associated with the production of acetate and butyrate [27-29]. Studies by Jiang *et al*. further emphasized the efficacy of strain DACNJS2 in alleviating BaP-induced nerve damage by enriching Lachnospiraceae and increasing butyrate levels. Additionally, an increase in Muribaculaceae abundance plays a significant role in neuroprotection [30]. While Oscillibacter (family Oscillospiraceae) was among the genera showing the most prominent differences in our analysis, its specific role in SCFA production and neuroprotection requires further investigation [31].

Various studies have demonstrated a connection between the brain and the gut microbiota, known as the gut-brain axis [32-35]. Additionally, Kang used germ-free (GF) and conventional C57BL/6 mice to induce liver cirrhosis through gavage with carbon tetrachloride (CCl4) and compared them with non-cirrhotic control groups. Conventional cirrhotic mice exhibited gut microbiota dysbiosis, systemic inflammation, and neuroinflammation, while GF cirrhotic mice only showed elevated ammonia levels without accompanying inflammation. This provides theoretical support for the notion that ECH influences neuroinflammation by modulating the gut microbiota [36]. The blood-brain barrier (BBB) was once believed to protect microglial activity, but it is now recognized that it is influenced by factors external to the gut and central nervous system [37].

Our research findings align with these previous findings, indicating that ECH treatment was associated with alterations in gut microbiota composition, which correlated with reduced neuroinflammatory markers, suggesting a potential link that warrants further mechanistic investigation. Furthermore, we also discovered that alterations in the gut microbiota may affect brain function and behavioral performance through pathways such as influencing metabolite production, synthesis and release of neurotransmitters, and regulation of the immune system. In a nested case-control study, researchers assessed emotional symptoms, plasma neurotransmitter levels (serotonin and norepinephrine), and gut microbiome characteristics in 40 patients with irritable bowel syndrome (IBS) and 20 healthy controls (HC). The results revealed significant differences between the IBS and HC groups in emotional distress and microbiome characteristics, although neurotransmitter levels were lower in the IBS group without reaching statistical significance. Further analysis showed a negative correlation between norepinephrine levels and gut microbial α-diversity within the IBS group, a positive correlation between serotonin levels and the abundance of Proteobacteria, and a positive correlation between norepinephrine and Bacteroidetes, with a negative correlation with Firmicutes. These findings are similar to our experimental results and those reported by Barandouzi [38]. This bidirectional interaction provides a more comprehensive perspective for understanding the neuroprotective effects of ECH.

Despite the significant progress made in our research, several limitations should be acknowledged. The genus-level comparisons from the 16S rRNA sequencing data were not corrected for multiple testing, which may increase the risk of false-positive findings; therefore, the observed differences should be interpreted as exploratory. Our assessment of neuroinflammation was primarily limited to Iba-1 immunostaining and TNF-α expression. While these markers are widely accepted indicators of microglial activation and pro-inflammatory cytokine production, they do not fully capture the complexity of the neuroinflammatory cascade. Additional inflammatory mediators, such as IL-1β, IL-6, COX-2, and NF-κB pathway components, were not examined in the current study. Future investigations incorporating these markers will be necessary to fully elucidate the anti-inflammatory mechanisms of ECH.

It should be noted that the LPS-induced acute neuroinflammation model, while widely used for studying the mechanisms of inflammation-associated cognitive impairment, does not fully recapitulate the chronic progressive pathology of neurodegenerative diseases such as Alzheimer’s disease or Parkinson’s disease. Therefore, the neuroprotective effects observed in the present study should be interpreted within the context of acute neuroinflammation. Further validation in chronic neurodegenerative disease models (*e.g.*, transgenic APP/PS1 mice or MPTP-induced Parkinson’s models) is warranted to explore the broader therapeutic potential of ECH.

We acknowledge that the sample size of six mice per group, while consistent with many previous LPS-induced neuroinflammation studies, may limit the statistical power for behavioral assessments such as the MWM, given the inherent variability in rodent spatial learning performance. Nevertheless, the robust and consistent differences observed across multiple independent behavioral parameters (escape latency, path length, target quadrant time, and platform crossings) support the validity of our findings. Future studies with larger cohorts would further strengthen the conclusions.

In summary, our study suggests that ECH exerts neuroprotective effects in the LPS-induced neuroinflammation model, accompanied by remodeling of the gut microbiota. These findings provide preliminary evidence for the potential of ECH in alleviating neuroinflammation-related cognitive impairments, though further validation in chronic neurodegenerative disease models is warranted.

## Conclusison

In conclusion, this study demonstrates that ECH, the main active component in the traditional Chinese medicine herb *C. tubulosa*, exerts neuroprotective effects in an LPS-induced neuroinflammation model by attenuating neuronal injury in the cerebral cortex and hippocampus, inhibiting the secretion of the inflammatory factor TNF-α, and modulating the gut microbiota structure. These effects collectively contribute to the improvement of LPS-induced cognitive impairment in mice. The study highlights the neuroprotective potential of ECH in the context of acute neuroinflammation and suggests that further investigation in chronic neurodegenerative disease models is warranted to fully evaluate its therapeutic potential.
